# Demetallization of Sewage Sludge Using Low-Cost Ionic Liquids

**DOI:** 10.1021/acs.est.0c03724

**Published:** 2021-03-16

**Authors:** Joseph G. Yao, Sze-yin Tan, Philip I. Metcalfe, Paul S. Fennell, Geoffrey H. Kelsall, Jason P. Hallett

**Affiliations:** † Department of Chemical Engineering, Imperial College London, South Kensington, Exhibition Road, London SW7 2AZ, United Kingdom; ‡ Efficiency Technologies, Bluecube House, Milton Keynes, Buckinghamshire MK12 5TS, United Kingdom

**Keywords:** Separation, electrodeposition, heavy metals, techno-economic evaluation, 1-methylimidazolium chloride, triethylammonium hydrogen sulfate, dimethylbutylammonium hydrogen sulfate

## Abstract

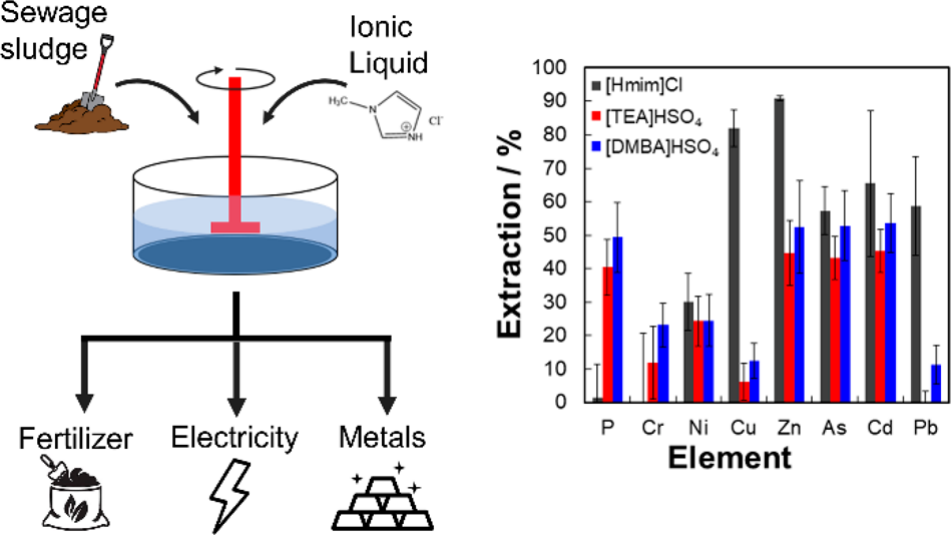

Sludge produced from wastewater treatment has little to no value and is typically treated through volume reduction techniques, such as dewatering, thickening, or digestion. However, these methods inherently increase heavy metal concentrations, which makes the sludge unsuitable for land spreading and difficult to dispose of, owing to strict legal requirements/regulations concerning these metals. We addressed this problem, for the first time, by using recyclable low-cost protic ionic liquids to complex these toxic metals through a chemical fractionation process. Sewage sludge samples collected from wastewater plants in the UK were heated with methylimidazolium chloride ([Hmim]Cl, triethylammonium hydrogen sulfate ([TEA][HSO_4_]) and dimethylbutylammonium hydrogen sulfate ([DMBA][HSO_4_]) under various operating temperatures, times and solids loadings to separate the sludge from its metal contaminants. Analysis of the residual solid product and metal-rich ionic liquid liquor using inductively coupled plasma-emission spectrometry showed that [Hmim]Cl extracted >90% of Cd^II^, Ni^II^, Zn^II^, and Pb^II^ without altering the phosphorus content, while other toxic metals such as Cr^III^, Cr^VI^ and As^III^ were more readily removed (>80%) with [TEA][HSO_4_]. We test the recyclability of [Hmim]Cl, showing insignificant efficiency losses over 6 cycles and discuss the possibilities of using electrochemical deposition to prevent the buildup of metal in the IL. This approach opens up new avenues for sewage sludge valorization, including potential applications in emulsion fuels or fertilizer development, accessed by techno-economic analysis.

## Introduction

Owing to rapidly increasing global population and urbanization, there has been a significant increase in wastewater production.^1^ Despite being composed of mostly water (ca. < 0.1% solid material), wastewater can cause significant damage to the environment. Except in the most highly developed countries, wastewater is released directly to the environment without suitable treatment, resulting in detrimental impacts on humans and the quality of freshwater resources and ecosystems.^2,3^ However, treatment of wastewater also comes with a price: production of significant quantities of sewage sludge, a semisolid made up of residual organic matter and dead bacteria^3^ which is often difficult to treat and dispose of.^4^ Since it is rich in organic matter and nutrients, there are considerable reuse opportunities as a soil conditioner and a fertilizer, but this potential is often not realized owing to the presence of pathogens and heavy metals.^5^ Heavy metals such as Zn^II^, Cu^II^, Ni^II^, Pb^II^, Cd^II^, Cr^III^, Cr^VI^, Hg^II^, As^III^, and As^VI^ are particularly hazardous^6,7^ and, due to their high solubility in aquatic environments, may end up accumulating in the human body via the food chain.^6^ Although Zn^II^ and Cu^II^ are essential for humans, animals, and plants, larger amounts can be toxic.^8^ Ni^II^, Pb^II^, Cd^II^, Cr^II^, Hg^II^, As^III^, and As^VI^ are strictly toxic to the human body.^1,9^ Hence, research into metal removal techniques from wastewater and sewage sludge has been reported.^1,10^

For sewage sludge, heavy metals can be removed via biological^11,12^ and chemical extraction.^10,13^ Biological extraction, or bioleaching, uses chemolithotropic bacteria to oxidize metals into ions but is typically very slow, requiring at least 24 h to achieve high levels of extraction.^14^ Moreover, bioleaching requires stringent control over operating conditions, particularly the pH.^15^ Chemical extraction is generally much faster and can be achieved with acid treatment (H_2_SO_4_, HCl, HNO_3_, citric, and oxalic acid)^16^ or by contacting the sludge with chelating agents (EDTA/NTA).^17^ During acid treatment, the heavy metals in the sludge are dissolved via an exchange of protons (eq 1),^4^ while extraction with chelating agents forms an EDTA/NTA-metal complex (eq 2).^10^
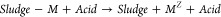
1
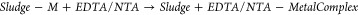
2

Although inorganic acid treatment can achieve up to 100% extraction of some metals at very low pH (1.5–2), they are highly corrosive in nature making the use of acid-resistant materials for reactor construction necessary, and the end product must be treated with large quantities of lime or an equivalent to neutralize its pH.^10^ Moreover, inorganic acids are not biodegradable, so the extraction process is not environmentally attractive, as is for chelating agents. Organic acids are biodegradable, but some (citric acid) can also achieve high levels of extraction. Unfortunately, organic acid treatment is slow and requires several days before acceptable levels can be reached and the final product must still be neutralized to precipitate the metals as hydrous oxides.^18^

The use of ionic liquids (ILs) offers a more sustainable chemical extraction technique to remove heavy metals from sewage sludge via a more environmentally benign process with fast extraction times and no neutralization requirements of the final product. ILs are salts composed of bulky, unsymmetrical organic cations with charge diffuse anions which are in a molten state at room temperature. To date, ILs have already been extensively studied for lignocellulose pretreatment in the context of biorefining and biofuel production^19−22^ but they have also been shown to be effective in the metal decontamination of waste wood^23,24^ and hydrocarbon streams.^25,26^ As a result of their ionic character, ILs have negligible vapor pressure under standard operating conditions, avoiding atmospheric losses, as well as good electrical conductivity, making them suitable for electrochemical applications such as electrodeposition.

For our work, we demonstrate, for the first time, the use of ultra-low-cost protic ionic liquids (PILs),^27^ which can be synthesized by an acid–base neutralization reaction, for the extraction of heavy metals from sewage sludge. Although ionic liquids have been studied for heavy metal removal from wastewater in the past,^28−30^ the focus was mainly on extracting heavy metals directly from wastewater rather than from sludge. Moreover, the ILs that have been studied to date are significantly more expensive to synthesize compared to that of the PILs used within this study. The toxicity of these PILs is expected to mimic that of their parent amine and acid. Shorter alkyl chains and less complex structures on the PILs lead to lower toxicities and good biodegradability.^20,31,32^ Here, we demonstrate that it is possible to extract metals chemically from sewage sludge, to extents comparable to acid treatment but with much shorter contact times. We focused on Zn^II^, Cu^II^, Ni^II^, Pb^II^, Cr^III^, Cr^VI^, and Cd^II^, as these are regulated when sludge is composted. In addition, As^III^ and As^VI^ were also monitored. We demonstrated that it is also possible to recover and reuse the IL in a recyclable and continuous manner, which has not been shown with other chemical extraction techniques, but this is vital for comprehensive process design. Furthermore, we explore the possibility of recovering dissolved metals from the IL-metal stream via electrodeposition.

Three types of ILs were used in this study: 1-methylimidazolium chloride ([Hmim]Cl), triethylammonium hydrogen sulfate ([TEA][HSO_4_]), and dimethylbutylammonium hydrogen sulfate ([DMBA][HSO_4_]). Recently, [Hmim]Cl was shown to successfully fractionate and decontaminate Cr^III^, Cu^II^, and As^IV^ from treated waste wood, with Cu^II^ extraction from the pulp reaching >98% while Cr^III^ and As^IV^ extraction fluctuated slightly between 60 and 80% and 80–95%, respectively,^24^ while [HSO_4_]-based ILs have been shown to have superior biomass deconstruction capabilities^22,33^ with Cr^III^, As^IV^, Ni^II^, and Cd^II^ extractions of >80% from waste wood fines.^23^

## Materials and Methods

### Ionic Liquid Synthesis

Three types of ILs were synthesized for these experiments: 1-methylimidazolium chloride ([Hmim]Cl), triethylammonium hydrogen sulfate ([TEA][HSO_4_]), and dimethylbutylammonium hydrogen sulfate ([DMBA][HSO_4_]) using established procedures, and the formation of the IL was verified using NMR spectroscopy; see the Supporting Information (SI) for further details.

### Sample Characterization

The sludge samples investigated in this work include Severn Trent sewage cake (STC) (Severn Trent, Minworth Water Treatment plant), Southern Water sewage cake (SWC) (Southern Water, Millbrook plant), and Southern Water digestate (SWD) (Southern Water, Millbrook plant). The Severn Trent and Southern Water sewage cake samples were dried overnight at 35 °C under air in an oven (Carbolite) and then crushed with an agate pestle and mortar. The Southern Water digestate was dried in a 4.5 FreeZone freeze-dryer (Labconco) for 48 h before oven drying at 35 °C and crushing. The final moisture content of the dry solids was determined using the NREL protocol for determining the solids content of biomass.^34^ The sludge (and the treated samples) were further characterized using ultimate and proximate analysis carried out on a Vario MICRO CUBE elemental analyzer (Elementar) and Q5000 IR TGA (TA Instruments), respectively. Further details are in the SI.

### Chemical Extraction with ILs

The dry solids were weighed out into 15 mL Ace pressure tubes (Sigma-Aldrich), and to each sample, a designated weight of ionic liquid was added, depending on the desired loading, but a typical solid/liquid (S/L) loading of 0.1 (1:10 ratio of oven-dried sample to IL by mass) was used. Other S/L values studied were 0.2, 0.5, and 1. The contents of the pressure tubes were then mixed using a vortex shaker (VWR International) for 1 min before they were transferred into an oven (Thermo Fisher Scientific) set to the desired operating temperature (room temperature, 30 °C, 60 °C, 90 or 120 °C). After a set time (15, 30, 45, or 60 min), the pressure tubes were removed from the oven and allowed to cool down under ambient conditions. The contents were then transferred into 50 mL falcon tubes using ca. 40 mL of ethanol as a solvent. The ethanol/IL liquor was separated from the solids using a centrifuge (VWR International) set at 3000 rpm for 45 min. The supernatant ethanol/IL mix was decanted from the falcon tubes into round-bottom flask before being topped up with fresh ethanol and then mixed and centrifuged again. After 4 rounds of ethanol washing, the solids were transferred into cellulose thimbles (VWR International) and washed with ethanol in a Soxhlet extractor. The solids in the thimble were air-dried overnight before being weighed and analyzed for metal content. This solids fraction is referred to as “Treated Fraction” in this paper. The ethanol from the Soxhlet was then combined with the other washings. The IL was separated from the ethanol using a rotary evaporator (Buchi) set to 9 kPa and 45 °C. Once the ethanol had evaporated, the IL was washed with water as a counter solvent to precipitate any ethanol-soluble material (denoted “Residue”). The IL was then recovered by evaporating the water at 4 kPa and 45 °C. The heavy-metal rich IL is referred to as “IL Liquor” in this paper. All experiments were conducted in triplicates per sample, and the average values are presented. The error was calculated based on the standard deviation value.

### Trace Element Analysis

The heavy metal content of the solid samples was determined by closed *aqua regia* microwave digestion using an adapted 3051a protocol (for sediments and sludges) followed by ICP-MS (Agilent Technologies). Further details can be found in the SI. A certified reference material, ERM 144 Sewage Sludge (trace elements), was digested with the same procedure in order to validate the results. The % recovery of the reference materials can be found in the SI (Figure S3).

### Electrodeposition and Solubility Studies

To determine the solubilities of the key metals, metal-IL solutions were prepared by slowly adding metal salts to aliquots containing 3 g of [Hmim]Cl. The samples mixed with a vortex mixer until they were oversaturated. The metal salts used in this study were K_3_PO_4_ (Sigma-Aldrich), CrCl_3_ (Fluka), NiCl_2_·2H_2_O (Alfa Aesar), CuCl_2_·2H_2_O (Alfa Aesar), ZnCl_2_ (Sigma-Aldrich), PbCl_2_ (Sigma-Aldrich), AsCl_3_ (Sigma-Aldrich), and CdCl_2_ (Sigma-Aldrich). These solutions were decanted into a separate container using a syringe and filter to remove any residual solids. The decanted samples were then diluted 500-times using a mixture of 2% HNO_3_ and 0.5% HCl, and their metal content was determined using ICP-MS. The results for the solubility studies can be found in the SI, Figure S4.

Electrochemical measurements were made using a potentiostat (AUTOLAB PGSTAT302F) and NOVA software in a three-electrode glass cell. A glassy carbon electrode (1.5 mm radius) was used as the working electrode for all electrochemical measurements. Before use, the working electrode was polished with a slurry of 50 nm alumina particles on a soft microfibre polishing pad (MicroCloth, Buehler Ltd.) and then on a clean wet microfibre pad. A platinum gauze and AgCl/Ag (3.0 M NaCl) acted as the counter and reference electrodes, respectively. The electrodeposition of Cu^0^, Pb^0^, and Cd^0^ in [Hmim]Cl were studied by chronoamperometry followed by linear scan voltammetry. The metal-IL solutions were prepared by doping 5 g of [Hmim]Cl (with 20 wt % H_2_O) to obtain 1000 ppm (μg g^–1^) concentration mixtures.

### Model Development

**Figure 1:**
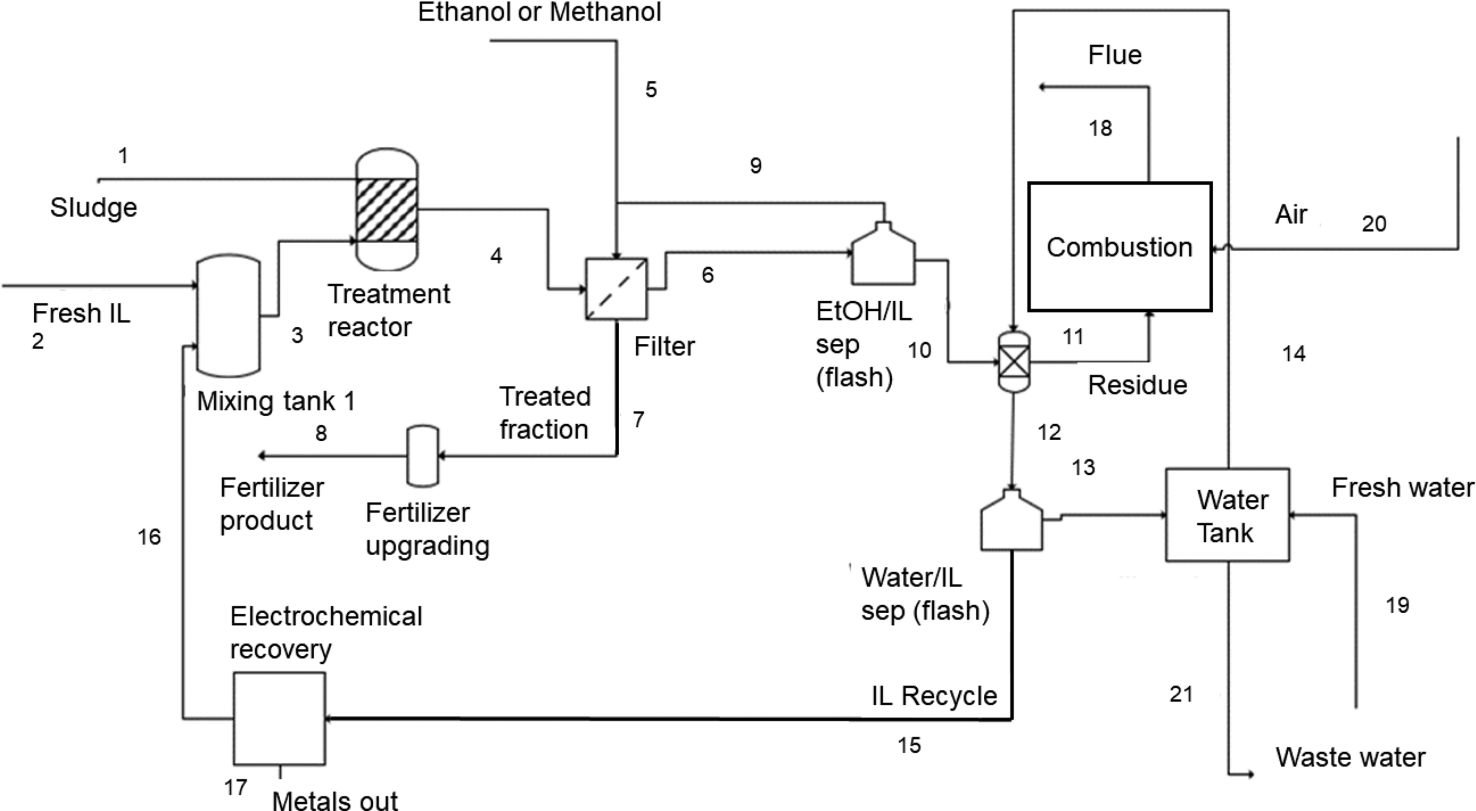
Process flow diagram for Pathway 1 (Pathway 2 is the same but without the electrochemical recovery block).

In order to assess the commercialization potential of the IL extraction process for sewage sludge treatment, a simple techno-economic assessment was carried out. Here, we estimated the capital and operating costs of seven different pathways using standard chemical engineering costing techniques.^35^ The pathways are highlighted in Table 1, and the schematic for Pathway 1 (the process that utilizes the most waste) is given by Figure 1. Other pathway schematics can be found in the SI (Figures S5–S7). For this model, we used our experimental results to determine the component mass balances, compositions, recoveries, energy content upon combustion, and recycle rates.

**Table 1:** Modelled Pathways and Their Key Products

Pathway	Product 1	Product 2	Product 3
1	Fertilizer (treated fraction)	Electrical energy (residue)	Recovered metals
2	Fertilizer (treated fraction)	Electrical energy (residue)	
3	Fertilizer (treated fraction)	Recovered metals	
4	Fertilizer (treated fraction)		
5	Fertilizer (ash)	Electrical energy (treated fraction)	Recovered metals
6	Fertilizer (ash)	Electrical energy (treated fraction)	
7	Electrical energy (treated fraction)		
8	Electricity (untreated sludge)		

For Pathway 1 (Figure 1), the model consists of a treatment reactor in which the sludge is contacted with a mixture of fresh and recycled IL, a filter to separate the solid fraction from the IL liquor after being washed with an organic solvent, a flash to remove the solvent for recycling; a contactor to remove the residue with water, a combustion chamber to burn the residue, a water storage tank, a second flash to separate the IL from the water, an electrodeposition reactor, and a mixing tank to recombine recycled IL with fresh IL. The moisture content of materials, the treated fraction yield, lignin yield, and IL recovery, which were required for determining the mass balance on the overall system, were determined using our experimental data.

The equipment installment costs were determined using the modular costing technique (eqs 3 and 4) and then deflated with the CEPCI index to the year 2017.
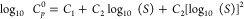
3
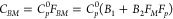
4where *S* is the capacity of the equipment and is determined using the mass balance model. *C*_p_^0^ is the purchased cost of equipment at the manufacturer’s site, *C*_BM_ is the bare module cost, and *F*_BM_ is the bare module factor. *C*_1_, *C*_2_, *B*_1_, *B*_2_ are equipment-dependent coefficients given by Turton.^36^*F*_P_ is the pressure factor, and *F*_M_ is the material cost factor. For each process unit that comes into contact with the IL, we selected borosilicate glass as the construction material owing to its high chemical resistance and reasonable cost.^37,38^ To estimate the material cost factor, we took the ratio of cost between borosilicate glass and carbon steel using material construction data^37^ and the fact that carbon steel has a material cost factor of 1.^39^ All other components were assumed to be fabricated from stainless steel 316.

For more general equipment, such as compressors, for which it was easier to obtain costs, the six-tenths rule (eq 5) was used. Here, *C*_1_ = capital cost of project with capacity *S*_1_, *C*_2_ = capital cost of project with capacity *P*_2_ and *n* is taken to be 0.6.
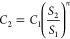
5

*C*_1_ and *S*_1_ were taken from previous work.^40^ The equipment capacities were calculated according to the mass flow rate of material and a residence time of 30 min for each unit operation. To determine the fixed capital cost, we used the factorial method^35^ assuming a fluid–solids system in which the storage and building infrastructure is already in place (i.e., the waste treatment plant) and that basic utilities such as cooling water were available from the plant in which this process would be built.

The cost of the ionic liquid reagents were taken from Alibaba (August 2018), while the price of electricity, gas, and water were taken from British Gas and Severn Trent tariffs for the cost-year.^41,42^ The specific electrical energy consumption (*w*^e^_P_) of electrodeposition of metal product *P* with molar mass *M*_P_ was calculated from eq 6 in which *v*_e_ represents the electron stoichiometry of the electrodeposition reaction, *F* is the Faraday constant,  is the charge yield, and *U* is the reactor operating potential difference
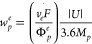
6

Hence, with eq 6 predicting the dominant component of the process running costs, together with the specific electrical energy cost ($ kW^–1^ h^–1^), the metal product specific cost can be estimated for comparison with prices (London Metal Exchange, www.lme.com; August 2018).^43^ The quantity of metals deposited were determined using the extraction efficiency measured by our experiments.

## Results and Discussion

**Figure 2:**
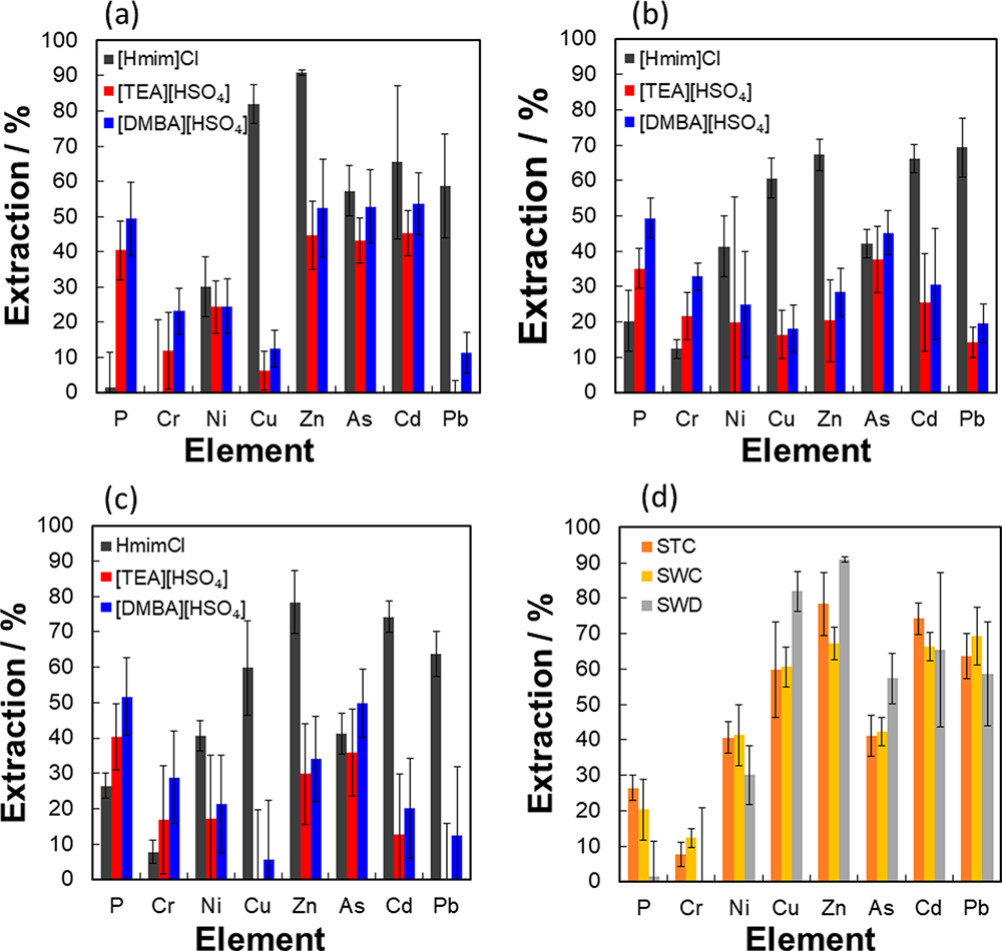
Metal extractions from treating (a) Southern Water digestate (SWD), (b) Southern Water cake (SWC), and (c) Severn Trent cake (STC) with three ILs. (d) A comparison of metal extractions from SWD, SWC, and STC using [Hmim]Cl. Treatments in all cases were conducted at 120 °C for 45 min with S/L = 0.1.

Figures 2a–d report the removal of metals after 0.75 h from each sewage sludge sample with each IL, with [Hmim]Cl demonstrating the best overall extraction (calculated according to eq 7) for five of the elements in the order of Zn^II^ > Cu^II^ > Cd^II^ > Pb^II^ > As^III^ > Cr^III^.
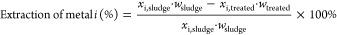
7

Here, *x*_*i,j*_ is the concentration of metal, *i* in *j*, and *w*_*,j*_ is the mass. Similar extraction efficiencies of As^III^ and As^V^ (ca. 50%) and Ni^II^ (20–30%) were also achieved by [TEA][HSO_4_] and [DMBA][HSO_4_]. Although these two ILs were marginally better at removing Cr^III^, they had little affinity for Pb^II^ and were found to extract a significant amount (40–50%) of the P^V^, a key component of fertilizers. Although the extraction efficiencies differed between the treated sludges in % terms (Figure 2a–c), a single IL will preferentially extract the same element over the others (i.e., [Hmim]Cl showed greater affinity to Zn^II^, Cu^II^, Ni^II^, Pb^II^ for all three sludge samples). This result is more clearly highlighted in Figure 2d. Although the extraction rates for Pb^II^, Cd^II^, Cr^III^, Cu^II^, Ni^II^, and Zn^II^ were found to be lower than those achieved using the ILs, trihexyl(tetradecyl)phosphonium 2-(methylthio)benzoate ([PR_4_][TS]) and trihexyl(tetradecyl)phosphonium thiosalicylate ([PR_4_][MTBA]),^28^ where ≥90% removal was achieved from real wastewater and activated sewage sludge samples, our protic ILs were synthesized using cheaper reagents and a simpler process.^44^

Other ILs (i.e., 1-hexyl-3-methylimidazolium hexafluorophosphate ([Hmim][PF_6_]) and 1-octyl-3-methyl-imidazolium hexafluorophosphate ([Omim][PF_6_])) have achieved extractions of ca. 90% from aqueous Cr^III^ and Cr^VI^ solutions.^45^ PbSO_4_ is very insoluble,^46^ so [HSO_4_]^−^ ILs would be expected to achieve lower Pb^II^ extraction efficiencies compared to Cl^–^ ILs. Similarly, Zn^II^ and Cu^II^ chlorides are also much more soluble than their sulfate counterparts,^47^ so high levels of extraction were obtained. The relationship between solubility and extraction is supported by a report^48^ demonstrating that increasing the solubility of metal ions by adding HCl improved their Cu^II^ extraction. Also, the authors argue that the metals form a complex with the IL during extraction, suggesting the anion species plays a key role in the extraction. This is evidenced by our work here, where we found a greater difference in extraction efficiency between Cl^–^ and [HSO_4_]^−^ ILs than between [TEA]^+^ and [DMBA]^+^ ILs. This anion influence is also observed by de los Ríos et al.^49^ who only saw changes in levels of extraction when they altered the anion in their imidazolium and ammonium-based ILs. However, rather than a complex-formation mechanism, they suggested that the anion and metal ions form an ion pair.

In either case, it appears that the key driver behind the high levels of extraction can be attributed to the Cl^–^ ions. Although there has yet to be any published work on the use of Cl^–^ based ILs for sewage sludge treatment, there have been cases where HCl was used directly. Wozniak and Huang^50^ found that chemical extraction with HCl was mainly influenced by the solids concentrations, the metal species, contact time, and pH. The metals were more readily solubilized under low pH conditions and 12 h of treatment. The best removal efficiencies with HCl were determined at pH 1 by Marchioretto et al.^51^ who reported extraction efficiencies of 100%, 80%, 80%, 100%, 60%, and 80% for Cd^II^, Cr^III^, Cu^II^, Pb^II^, Ni^II^, and Zn^II^, respectively, after 10 days of treatment. Interestingly, Cd^II^, Cu^II^, Pb^II^, and Zn^II^ were also the easiest to extract for our [Hmim]Cl IL giving comparable extraction efficiencies at considerably shorter contact times (45 min). In contrast, heavy metal extraction with H_2_SO_4_ has generally shown low extraction efficiencies for Cu^II^ and Pb^II^.^13,52,53^ Sylianou et al.^4^ have demonstrated up to 74% extraction for Ni^II^, 86% for Cu^II^, 99% for Cr^III^, Cr^VI^, 11% for Pb^II^, and 72% for Zn^II^ when they treated sludge samples from wastewater treatment plant in Athens, Greece with 20% H_2_SO_4_ for 30 min at 80 °C and a S/L ratio of 0.2, although the authors also comment on the considerable scatter among reported literature values. Silva et al.^54^ achieved similar levels of extractions when H_2_SO_4_ was used to contact sludge for 24 h at room temperature. Further to this, the extraction efficiency trends reported herein are in good agreement with previous reports where high metal extraction efficiencies from waste wood fines of ≥90% for Cd^II^, Cu^II^, Pb^II^, and Zn^II^ were achieved using [Hmim]Cl with exceptionally low extractions efficiencies determined for Cu^II^ and Pb^II^ using [Hmim][HSO_4_].^23^

Table 2 shows the change in metal ion concentration before and after the IL treatment process with [Hmim]Cl at 120 °C for 45 min with a S/L loading of 0.1. It can be seen that prior to treatment, the concentrations of Cu^II^, Zn^II^, and Cd^II^ in the Southern Water digestate and cake samples exceeded the allowable limits as prescribed by the PAS 100:2018^55^ guidelines, Publicly Available Specification for Composted Materials. However, after they are treated with [Hmim]Cl, the concentrations of these metal ions decreased to within acceptable values. Use of the same treatment conditions to clean up the heavy metals in the Severn Trent Cake, although significantly reducing the metal concentrations of Cu^II^, Zn^II^, and Cd^II^ to acceptable levels, failed to meet the guidelines as the Ni^II^ and Cr^III/VI^ contents remained above the PAS 100 threshold. This suggests the process requires further optimization, potentially involving sequential applications of IL to ensure that all sewage sludges are treatable.

**Table 2:** Metal Content of the Sludge Samples^a^

	Elemental concentration/mg (kg dry matter)^−1^							
Sludge sample	P^V^	As^V^	Cr^III^	Ni^II^	Cu^II^	Zn^II^	Cd^II^	Pb^II^
**STC**	(25800)	(8.3)	(244)	(127)	(357)	(1740)	(3.8)	(75.7)
	19000	4.9	225	75.6	144	375	1.0	27.4
**SWC**	(26400)	(4.3)	(49.0)	(23.9)	(470)	(533)	(1.8)	(52.5)
	19400	2.5	43.0	14.0	185	186	0.6	16.1
**SWD**	(23300)	(4.3)	(40.5)	(23.3)	(396)	(568)	(1.5)	(41.9)
	23000	1.8	40.5	16.3	71.3	48.4	0.5	17.3
**Treated STC+SWC**(30:70)	20400	3.2	97.6	32.5	173	243	0.7	19.5
**Treated STC+SWD**(30:70)	21800	2.8	95.9	34.1	93.0	146	0.7	20.3
**PAS 100 limits**			100	50	200	400	1.5	200
**Maximum permissible concentration in soil (pH > 5)**		50	400	50	80	200	3	300

a Southern Water digestate (SWD), Southern Water cake (SWC), and Severn Trent cake (STC) (before) and after treatment with [Hmim]Cl at 120 °C for 45 min with S/L = 0.1.

Table 3 shows the elemental, fixed, carbon, volatile, and ash content of the Severn Trent cake and the post-treatment products. The ash content remained unchanged between the untreated and treated fractions, suggesting that the IL did not extract any of the silicates/metals bound to the silicates. Moreover, the C, H, N, O, and S content also remained unchanged. In contrast, the residue fraction was found to be rich in carbon and low in ash, making it a potentially attractive fuel for power generation. The energy content of each component was determined using a high heating value (HHV/MJ kg^–1^) correlation (eq 8) for sewage sludge.^56^
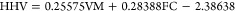
8where VM and FC represent the volatile matter and fixed carbon content, respectively. Although the energy content of the residue fraction appeared promising (being similar to that for woody biomass^57,58^), the yield of this fraction was only 10% relative to bulk sludge.

**Table 3:** Biomass Composition Determined through Ultimate and Proximate Analysis

	Ultimate Analysis/wt%^a^					Proximate Analysis/wt%			
Sludge fraction	C	H^c^	N	O^d^	S	Fixed Carbon^a^	Volatiles^a^	Ash^b^	HHV/MJ kg^–1^
**Severn Trent cake**	29.8	4.9	4.6	59.3	1.3	17.9	83.2	38.5	13.6
**Treated fraction**	28.7	4.1	4.2	62.0	1.0	23.2	76.7	38.7	13.7
**Residue**	56.4	8.1	3.5	25.3	6.8	14.7	84.8	0.60	20.5

a Dry, ash-free basis.

b Dry basis.

c Not including H in the moisture.

d Calculated by difference.

**Figure 3:**
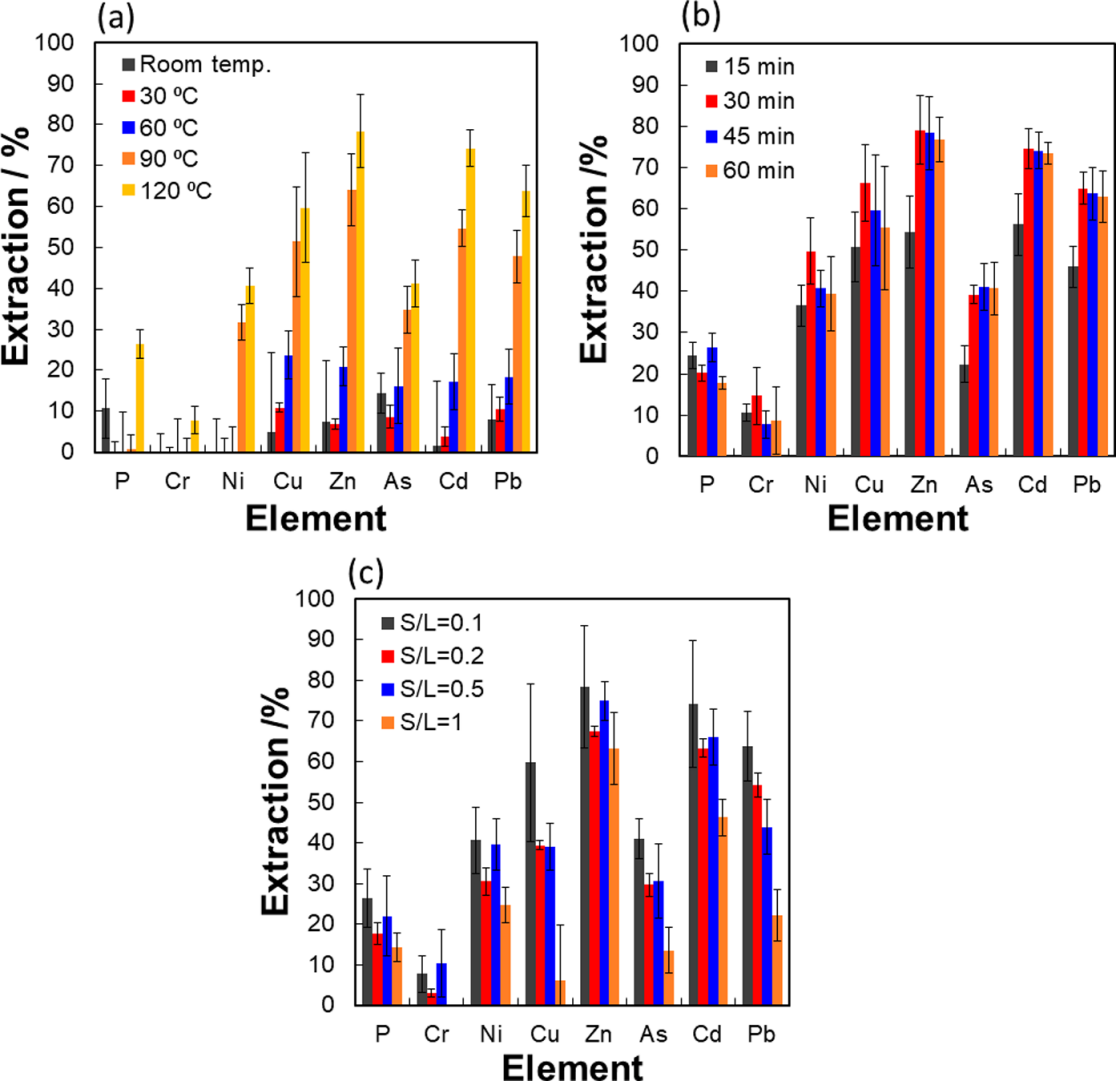
Metal extraction from (a) Severn Trent cake when treated with [Hmim]Cl for 45 min with S/L = 0.1 at temperatures 25–120 °C. (b) Severn Trent cake when treated with [Hmim]Cl at 120 °C for 15–60 min with S/L = 0.1. (c) Severn Trent Cake when treated with [Hmim]Cl at 120 °C for 45 min for S/L loadings from 1/10 to 1/1 (w/w).

In order to determine process conditions which could minimize energy consumption and equipment costs and reduce solvent consumption, it was necessary to determine the effects of reducing operating temperatures (from 120 °C down to room temperature), reactor residence times (from 60 to 15 min), and increased solid loadings (from 0.1 to 1 S/L ratios) (Figures 3a–c). Figure 3a suggests that there was a temperature threshold around 90 °C, below which any further decrease in temperature would result in a significant reduction (ca. 50%) in extraction efficiency, likely due to an increase in IL viscosity at lower temperatures decreasing mass transfer rates. Figure 3b shows that increasing contact times between the sludge and IL made little difference beyond 30 min, suggesting that the extraction kinetics were much faster than typical acid treatment and bioleaching processes.^15,16^ Although the solid loading had only a minor influence on extraction rates for low S/L ratios, increasing the solid-to-liquid ratio (Figure 3c) from 1:2 to 1:1 w/w ratio was found to have a marked effect on extraction efficiencies (ca. 50% decrease). For these conditions, the volume occupied by the solids was found to be much greater than the volume occupied by the liquids. Despite mixing the samples intensively with a vortex shaker, there was still suboptimal contact between the IL and sludge.

**Figure 4:**
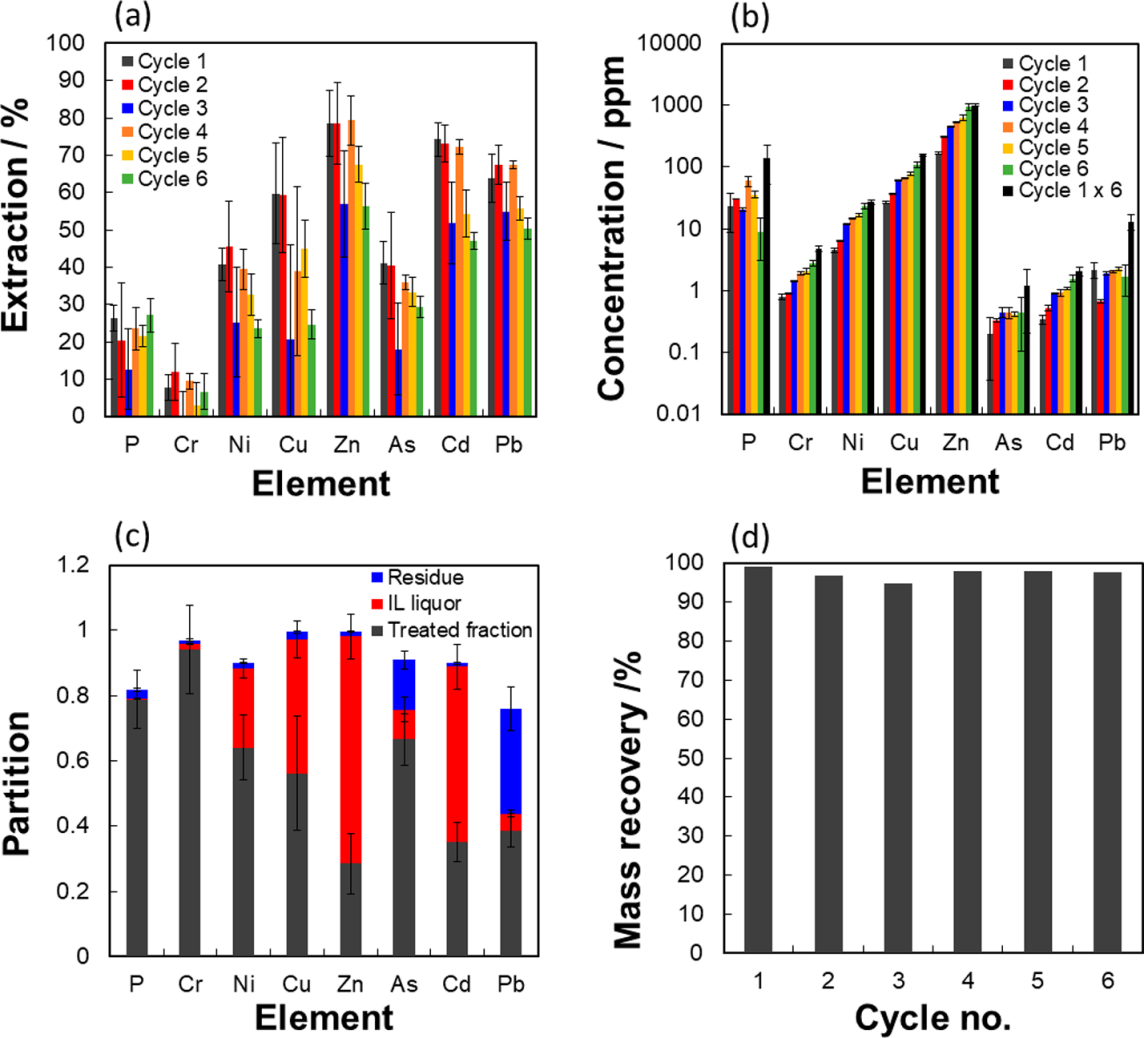
(a) Extraction performance of recycled [Hmim]Cl on Severn Trent Cake over 6 cycles, (b) metal ion accumulation within the IL liquor over 6 cycles, (c) overall partition of metal ions in the treated fraction, IL liquor and residue over the 6 cycles, and (d) mass recovery of IL over 6 cycles. Treatment was conducted at 120 °C for 45 min with S/L = 0.1.

To help determine the viability of this process, we also conducted experiments to test the recyclability of the IL. Figure 4a illustrates the change in extraction efficiency over six cycles of regenerating and reusing the IL. In each cycle, the metal ion-rich IL was recovered and corrected for its water content by evaporation/water addition before it was reused to extract metals from fresh Severn Trent cake. The results showed only minor losses of efficiency with each cycle. These findings are also reflected in Figure 4b, which shows a stepwise and consistent increase in heavy metal ion accumulation within the collected IL: the IL appeared to take up similar quantities of the same metal ion in each cycle. The accumulation of metal ions in the IL liquor is well within the solubility limits of [Hmim]Cl as shown in Figure SI4.

Analysis of its metal content makeup suggests that a significant portion of Pb^II^ and As^III^ partitioned into the residue. Here, the partition of heavy metal ions into the resultant fractions are defined according to eqs 9–11
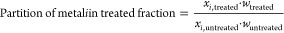
9
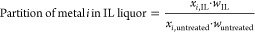
10
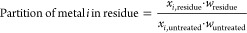
11

The concentration (w/w) of metal species *i* is denoted as *x*_*i*_, and the mass of the material is represented by *w*. The partitioning of the metal ions also shows that most metal ions, as expected, passed into the IL phase. Figure 4c shows that the partitioning of metal ions into the three phases remained unchanged relative to that of the first cycle (Supporting Information) and was also very consistent across each cycle. To our knowledge, this is also the first reported case of recycling ILs for sludge treatment.^59^ The mass balance (given by Figure 4d) demonstrates that there was no significant loss of IL during recycling. However, for the recovery of the IL, it is necessary to remove the metal ions with separation techniques such as ion-exchange, solid absorbents, or electrodeposition.^11^

**Figure 5:**
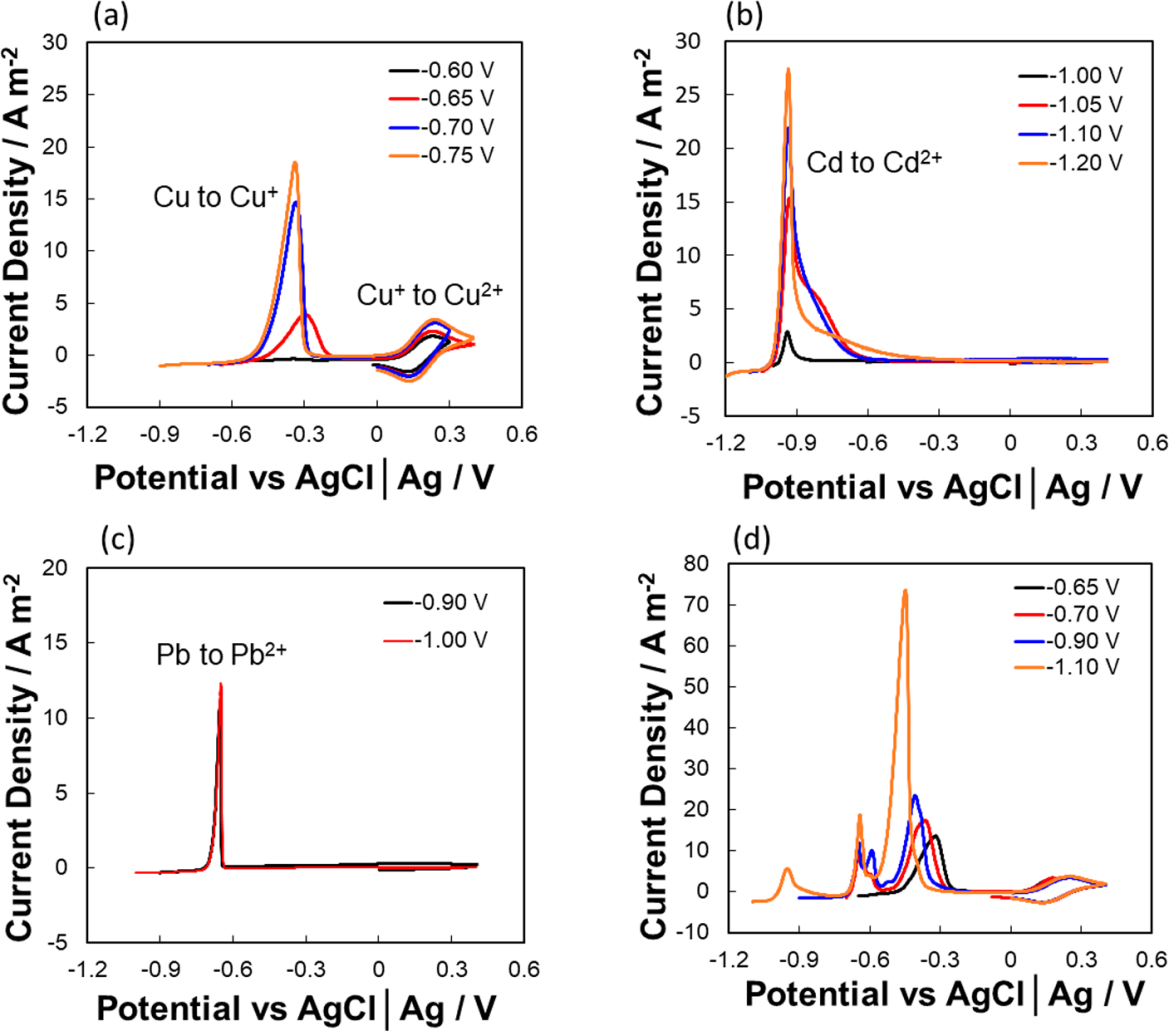
Anodic stripping voltammetry (10 mV s^–1^) following chronoamperometric electrodeposition (200s) of (a) Cu^II^, (b) Cd^II^, (d) Pb^II^ and (b) a mixture of Cd^II^, Pb^II^, Cu^II^ from 1000 ppm solutions of the correspond metals in [Hmim]Cl onto a glassy carbon electrode.

For the IL to be used in a recyclable manner, the accumulation of metal ions in the IL stream needs to be managed. ILs are attractive solvents for electrodeposition owing to their wide electrode potential window and high conductivities. Figure 5a–c illustrates voltammetric stripping of Cd^0^, Pb^0^, and Cu^0^ from [Hmim]Cl, respectively. For each solution of 1000 ppm concentration, electrodeposition occurred for a period of 200 s at a reducing potential followed by anodic stripping voltammetry at 10 mV s^–1^. As seen in Figure 5a, at an applied potential of −0.6 V vs AgCl|Ag, no significant peak was evident on the stripping voltammograms at ca. – 0.35 V. As more reducing potentials are applied from −0.65 to −0.75 V, increasing Cu stripping peak currents are observed. At applied potentials more negative than −0.75 V vs AgCl|Ag, no change in Cu stripping peak currents and charge was detected, implying that at such potentials, deposition occurred at a diffusion-limited rate. The presence of chloride ions stabilizes the Cu^I^ species in solution, which has been reported in aqueous chloride solutions.^60−62^Figure 5a shows quasi-reversibility of Cu^II^/Cu^I^ in [Hmim]Cl, with a peak-to-peak potential separation of 160 mV was observed for the one-electron transfer process. It can also be seen from Figure 5b,c that Cd^II^ and Pb^II^ deposited at diffusion-controlled rates at potentials greater than (−)1.05 V and (−)0.9 V, respectively.

Apparent charge yields for electrodeposition of Cu^0^, Pb^0^, and Cd^0^ are summarized in Table S1. For Cu^0^, the highest value of 0.79 occurred at −0.70 V vs AgCl|Ag. At less negative potentials, charge yields decreased due to kinetic limitations while more negative potentials resulted in increased rates of hydrogen evolution, also decreasing charge yields at the Cu-film substrate.^63^ The charge yield for electrodeposition of Pb^0^ was very low, as expected, since the deposition potentials of Pb^0^ overlap with the reduction of dissolved oxygen seen in the solvent window of metal free-[Hmim]Cl at ca. – 0.9 V (Supporting Information). The electro-generated superoxide species was unstable due to the presence of H_2_O in the IL and no oxidation reaction being detected in the voltammogram.^64,65^ The apparently higher charge yields observed from the deposition of Cd^II^ was due to the relatively higher concentration (mol L^–1^) of Cd^II^ compared to Pb^II^ in the 1000 ppm solutions. At higher Cd^II^ deposition potentials, the efficiency values decrease due to competition from hydrogen evolution reactions.

Due to the difference in electrodeposition potentials between Cu^II^, Pb^II^, and Cd^II^, electrodeposition at varying potentials was carried out from a mixed Cu^II^, Pb^II^, and Cd^II^ solution for 200 s. It is predicted that Cu^II^ can be recovered alone at potentials < −0.65 V vs AgCl|Ag, evidenced by a single anodic stripping peak in the voltammogram shown in Figure 5d. At–0.70 and −0.90 V vs AgCl|Ag, Pb–Cu alloys were deposited. Three anodic stripping peaks are observed, where peak 1 is associated with Cu stripping, while peaks 2 and 3 are associated with stripping of Pb deposited from glassy carbon and copper, respectively,^66^ as previously reported. At −1.10 V deposition, an additional peak 4 was evident due to prior electrodeposition of an Cd–Pb–Cu alloy. Further work is required to enable understanding of how the metals can be further separated from one another to produce high purity metals and how differences in metal concentrations affect the composition of alloy products.

### Techno-Economic Evaluation

Table S2 summarizes the main findings of our techno-economic study. Of the eight cases studied (see Table 1), Cases 5 and 6 are predicted to generate the most revenue if the selling price of the fertilizer is not included. This revenue is largely due to selling heat produced by burning the treated fraction. Here, we assume a unit price per kW h (Aug 2018) equal to that of UK gas suppliers. Since the gravimetric yield of the treated fraction is much greater than the residue, Cases 5–7 can generate more revenue compared to Cases 1 and 2. Our findings also demonstrate that although selling the recovered metals can be an additional source of revenue, it is not economical to sell them as the sole products. Moreover, the metals provide less economic value than the heat produced from combusting the treated fraction. To determine the feasibility of the different case scenarios, we calculated the required fertilizer selling price to achieve a positive net present value (NPV) over a period of 20 years assuming a discount rate of 13%. Our results show that for cases 1 and 2, the fertilizer must be sold at GBP_2017_ 280 and 243, respectively. Although this price reflects those of the current fertilizer market value (GBP_2017_ 158–330),^67^ the fertilizer produced from the treatment process will most likely need to be upgraded in phosphorus content to match the corresponding product specifications.^68,69^ Interestingly, if we eliminate the combustion part of the whole process, the necessary selling price of fertilizer reduces to GBP_2017_ 74–79 (Case 3 and 4). This is mainly due to a reduction in the total number of unit operations and therefore the capital cost. This also highlights the fact that the combustion is not economically self-sufficient. If there are subsidies or help to pay off the capital, then the required selling price can be further reduced. However, even with subsidies, if no fertilizer (or an equivalent product) is sold (Cases 6 and 7), then the process will fail to make a return on investment even after 20 years. After a comparison of each case to the base case scenario (Case 8, sludge incineration), Cases 1–5 are all potentially more economically attractive given the right fertilizer selling price.

## Supporting Information Available

Experimental details for ionic liquid synthesis, sample characterization, trace element analysis; figures and tables showing solubility limits of heavy metals in [Hmim]Cl, process flow diagrams, partitioning of elements extracted from STC, charge yields from electrodeposition, electrochemical solvent window of [Hmim]Cl, and details from techno-economic analysis of the IL treatment process (PDF)
