# Supplementary material for: Demetallization of Sewage Sludge Using Low-Cost Ionic Liquids
Source: Environ Sci Technol. 2021 Mar 16;55(8):5291–300. doi: 10.1021/acs.est.0c03724 (PMC8154363; doi:10.1021/acs.est.0c03724)
Supplement: Supplementary file 1 — es0c03724_si_001.pdf [file es0c03724_si_001.pdf]

Supporting Information: Demetallization of sewage sludge using low-cost ionic liquids

*Joseph G. Yao,† Sze-yin Tan,† Philip I. Metcalfe,‡ Paul S. Fennell,† Geoffrey H. Kelsall,† and Jason P. Hallett†,**

† Department of Chemical Engineering, Imperial College London, South Kensington, Exhibition Road London, SW7 2AZ, United Kingdom.

‡ Efficiency Technologies, Bluecube House, Milton Keynes, MK12 5TS, United Kingdom.

* Corresponding author: j.hallett@imperial.ac.uk

Number of pages: 13

Number of figures: 9

Number of tables: 5

Additional Experimental Details

Ionic Liquid Synthesis

Three types of ILs were synthesized for these experiments: 1-methylimidazolium chloride ([Hmim]Cl), triethylammonium hydrogen sulfate ([TEA][HSO_4]) and dimethylbutylammonium hydrogen sulfate ([DMBA][HSO_4]) shown in Figure SI1.

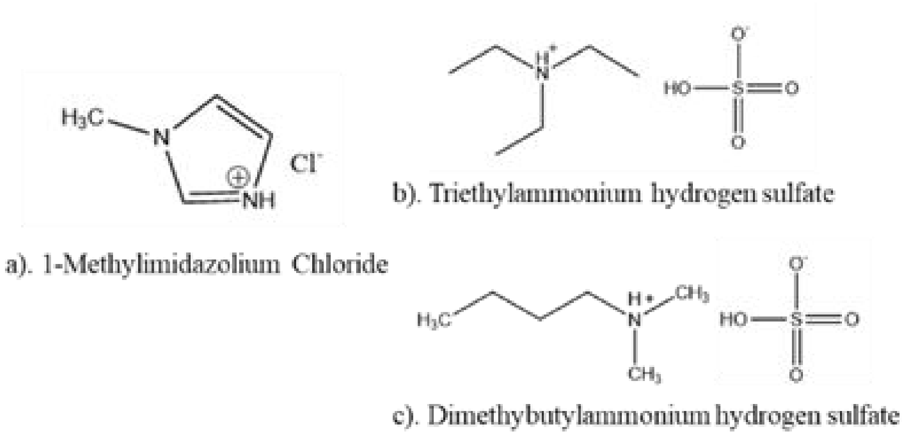

Figure S1: Chemical structures of the protic ionic liquids used in this study.

In each case, a pre-weighed quantity of acid (1 M for HCl and 5 M for H_2SO_4 (Sigma-Aldrich)) was added to an equimolar quantity of amine (Sigma-Aldrich) inside a round bottomed-flask using a funnel. The reacting solution was cooled constantly by submerging the round-bottomed flask inside an ice-water bath. The acid:base ratio was determined using a mass balance and confirmed by titration with 1 M NaOH using

a G20 compact titrator (Mettler Toledo). The water content was controlled using water addition/evaporation and was confirmed using a Karl Fischer titrator (Mettler Toledo). The formation of the IL was verified using NMR spectroscopy (Figure SI2).

a)

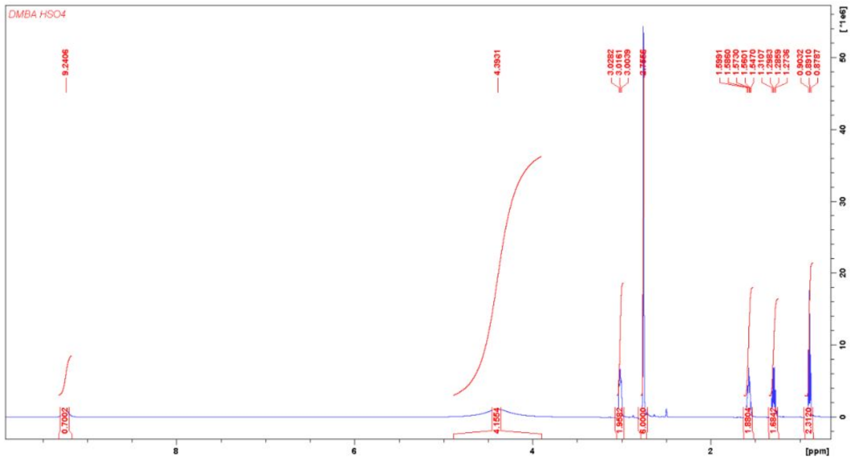

b)

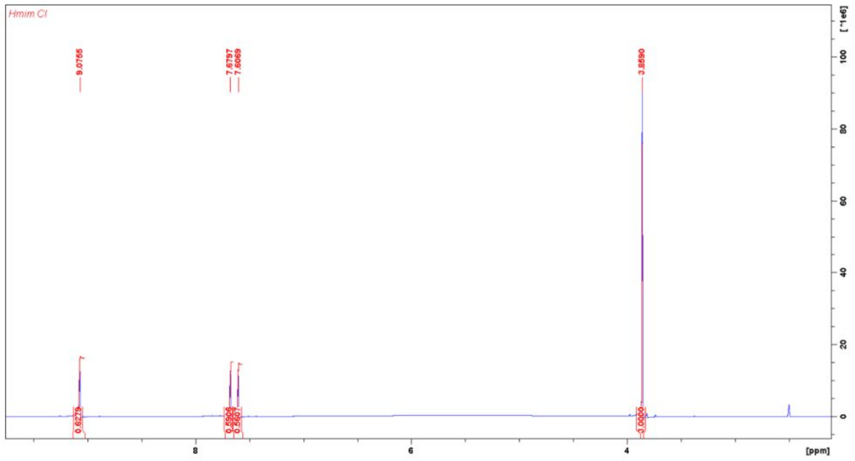

c)

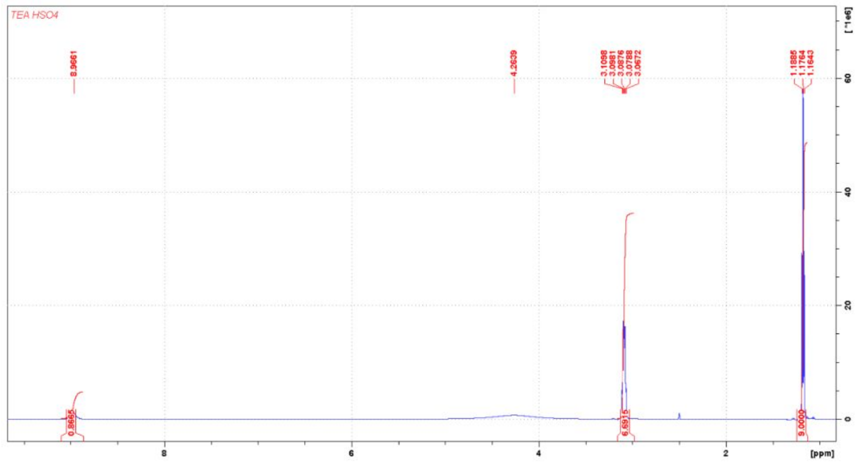

Figure S2: NMR spectra of [DMBA][HSO_4] (a), [Hmim]Cl (b) and [TEA][HSO_4] (c).

Sample Characterization

The sludge (and the treated samples) were further characterized using ultimate and proximate analysis carried out on a Vario MICRO CUBE elemental analyzer (Elementar) and Q5000 IR TGA (TA instruments), respectively. For the proximate analysis, the sludge samples were heated at a ramp rate of 60 °C min^{-1} under N_2 (50 ml min^{-1}) to 110 °C. After maintaining this temperature for 20 minutes to remove moisture, the sample was heated to 600 °C at a ramp rate of 30 °C min^{-1} and kept isothermal for another 20 minutes to remove the volatiles. The gas was then switched to a mixture containing 10 vol% O_2 (balanced by N_2) for 30 minutes to combust the carbon.

Trace Element Analysis

The heavy metal content of the solid samples was determined by closed *aqua regia* microwave digestion followed by ICP-MS. For each sample, 600 µL 67-69 % HNO_3 (trace element grade, Fischer Scientific) and 200 µL of 35-37 % HCl (trace element grade, Fischer Scientific) were added to 20 mg of the solid inside 20 mL MARS Xpress PFA vessels and left at room temperature overnight before being microwaved (MARS Xpress) using an adapted 3051a protocol (for sediments and sludges) whereby the samples were heated to 180 °C and held for 10 min before cooling down. The vessels were then transferred to a fridge for 1 h to aid cooling. After cooling, the digested samples were transferred into plastic volumetric flasks and made up to 10 mL using a 2 % HNO_3, 0.5 % HCl mix solution (prepared with ultra-pure 18 MΩ Milli-Q® water). The flasks where then shaken to facilitate mixing and passed through a 0.45 µm Whatman syringe filter. Acid digestion of the ionic liquors were carried out with the same procedure, but with conc. HNO_3 in a 1:10 v/v ratio (100 µL of liquid to 1 mL of HNO_3). After dilution, the vessels and volumetric flasks were rinsed with 5 % HNO_3, wiped with a tissue and submerged in a 5 % HNO_3 storage bath. The diluted samples

were analyzed using ICP-MS (Agilent Technologies) under helium and calibrated against solutions prepared from mixing and diluting single-element and multi-element standards (Inorganic Ventures) of known concentrations. The matrix effects of the dilution mixture were corrected using Sc^{II} (for P^{V}, Ni^{II}, Cu^{II}, Zn^{II}), Ge^{IV} (for As^{V}), Y^{III} (for Pb^{II}), and In^{III} (for Cd^{II}) as the internal standards. A certified reference material, ERM® 144 Sewage Sludge (trace elements), was digested with the same procedure in order to validate the results. The % recovery of the reference materials is shown in Figure SI3.

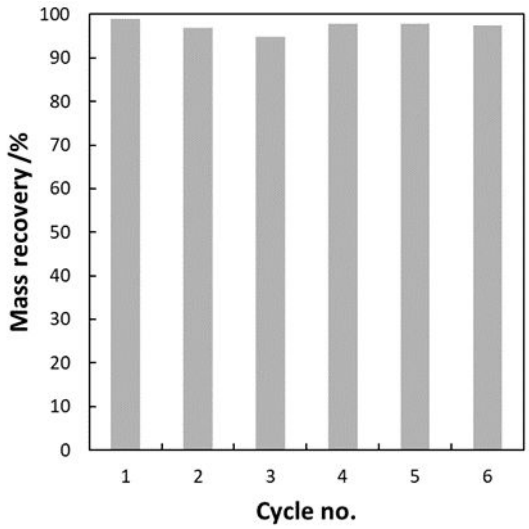

Figure S3: Metal recovery of certified reference material when 20 mg of the sample is digested in aqua regia using the protocol outlined in the main text.

Additional Figures and Tables

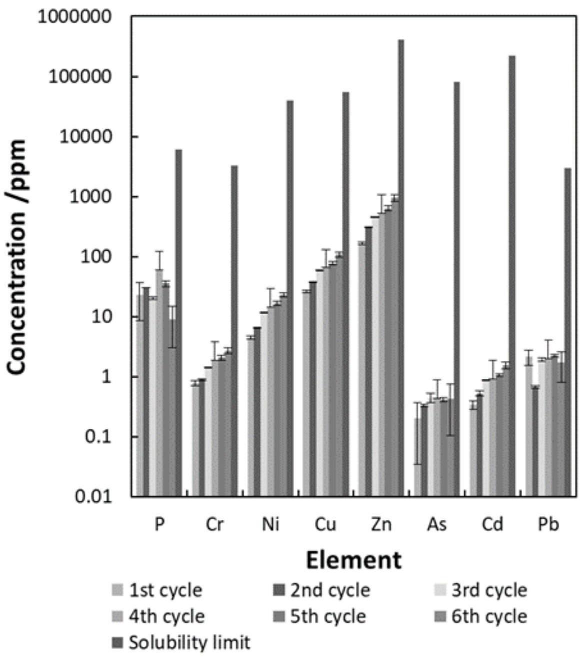

Figure S4: Concentration of heavy metals in recovered IL liquor (from Severn Trent Cake in [Hmim]Cl at 120 °C for 45 min with S/L=0.1 at 120) over 6 recycles versus their solubility limit in [Hmim]Cl.

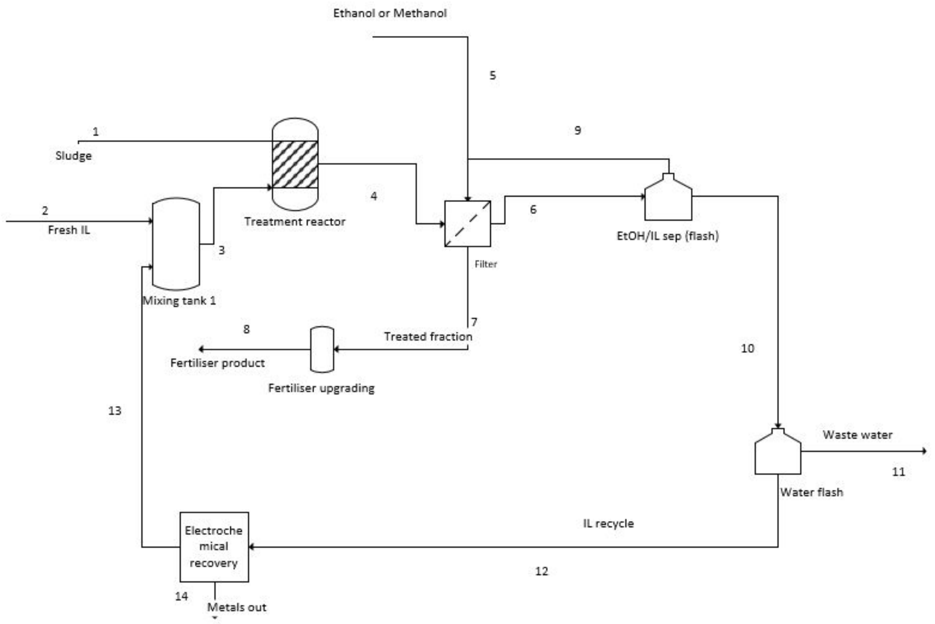

Figure S5: Process flow diagram for Pathway 3. (Pathway 4 is the same but without the electrochemical recovery block).

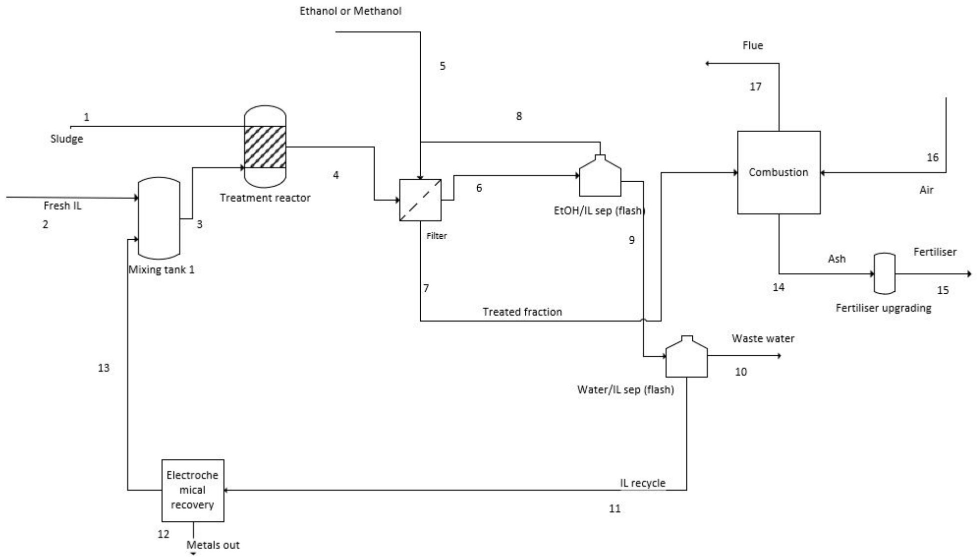

Figure S6: Process flow diagram for Pathway 5. (Pathway 6 is the same but without the fertilizer upgrading block. Pathway 7 is the same minus the electrochemical recovery and fertilizer upgrading block).

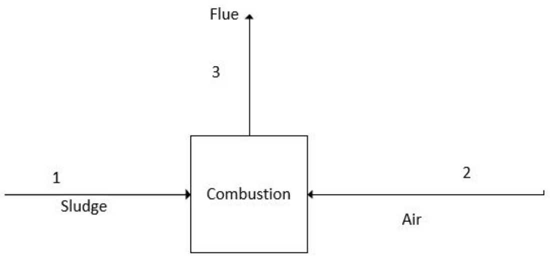

Figure S7: Process flow diagram for Pathway 8.

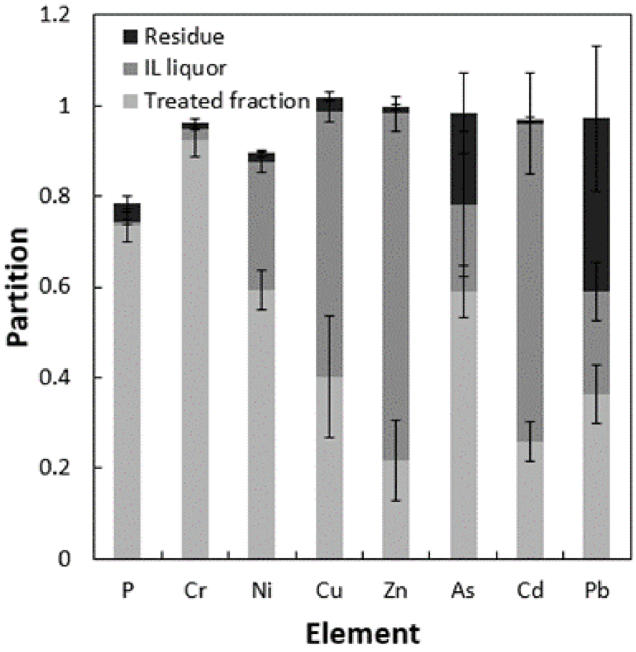

Figure S8: Partitioning of elements inside the treated fraction, ionic liquid and residue from extraction of Severn Trent cake treated with [Hmim]Cl at 120 °C for 45 min with S/L=0.1.

Table S1. Charge yields for electrodeposition from 1000 ppm solutions of Cu^{II}, Pb^{II} and Cd^{II} in [Hmim]Cl.

	**Deposition Potential vs. AgCl|Ag / V**	**Charge yield / 1**
**Cu**	-0.65	0.398
	-0.70	0.788
	-0.80	0.696
	-0.90	0.663
**Pb**	-0.90	0.240
	-1.00	0.190
**Cd**	-1.00	0.244
	-1.05	0.730
	-1.10	0.678
	-1.20	0.560

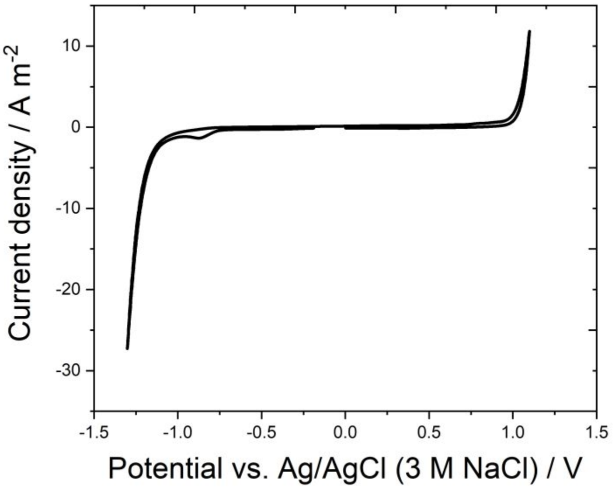

Figure S9: Cyclic voltammogram of glassy carbon electrode | [Hmim]Cl at 50 mV s^{-1}.

Table S2: Revenue and cost outputs from economic assessment of IL treatment process.

**Revenue and Cost** (GBP$_{2017}$ per tonne of sludge)	Case 1	Case 2	Case 3	Case 4	Case 5	Case 6	Case 7	Case 8
Copper	3	0	3	0	3	3	0	0
Zinc	2	0	2	0	2	2	0	0
Nickel	5	0	5	0	5	5	0	0
Lead	1	0	1	0	1	1	0	0
Chromium	5	0	5	0	5	5	0	0
Heat from untreated sludge	0	0	0	0	0	0	0	36
Heat from treated fraction	0	0	0	0	29	29	29	0
Heat from residue	4	4	0	0	0	0	0	0
Asset scrap value	7	6	1	1	6	5	3	9
**REVENUE less fertilizer price**	**26**	**9**	**17**	**1**	**51**	**50**	**32**	**45**
IL	17	17	17	17	17	17	17	£0
Sludge feed	0	0	0	0	0	0	0	0
Water evaporation	13	13	13	13	13	13	13	0

Ethanol evaporation	7	7	7	7	7	7	7	0
Electrodeposition	0	0	0	0	0	0	0	0
Scaled CapEX	0-70	0-56	0-14	0-11	0-60	0-60	0-28	85
**ANNUALISED COST**	**37-107**	**37-93**	**37-51**	**37-48**	**37-97**	**37-85**	**37-65**	**85**
**Fertilizer price UB***	**280**	**243**	**74**	**79**	**218**	**-†**	**-†**	
**Fertilizer price LB****	**210**	**187**	**60**	**68**	**158**	**-†**	**-†**	

*Upper bound estimate of fertilizer selling price required to breakeven after 20 years assuming discount rate of 13%. ** Lower bound estimate of fertilizer selling price required to breakeven after 20 years assuming discount rate of 13%. †No fertilizer is sold in this case scenario

Table S3: P-values from t-test performed on data from Figure 2 (main text). Metal extractions from treating (a) Southern Water digestate (SWD), (b) Southern Water cake (SWC) and (c) Severn Trent cake (STC) with three ILs. (d) A comparison of metal extractions from SWD, SWC and STC using [Hmim]Cl (d). Treatment in all cases were conducted at 120 °C for 45 min with S/L=0.1

	**Variable**	**Metals**							
(a)	IL	P	Cr	Ni	Cu	Zn	As	Cd	Pb
	[Hmim]Cl / [TEA][HSO_4]	0.007	0.430	0.428	0.001	0.001	0.064	0.199	0.003
	[Hmim]Cl / [DMBA][HSO_4]	0.004	0.140	0.447	0.001	0.008	0.568	0.434	0.006
	[TEA][HSO_4] / [DMBA][HSO_4]	0.307	0.201	0.982	0.229	0.472	0.247	0.250	0.055
(b)	IL	P	Cr	Ni	Cu	Zn	As	Cd	Pb

	[Hmim]Cl / [TEA][HSO_4]	0.068	0.090	0.367	0.001	0.003	0.491	0.008	0.001
	[Hmim]Cl / [DMBA][HSO_4]	0.008	0.002	0.172	0.001	0.001	0.526	0.019	0.001
	[TEA][HSO_4] / [DMBA][HSO_4]	0.038	0.065	0.836	0.774	0.363	0.317	0.690	0.254
(c)	IL	P	Cr	Ni	Cu	Zn	As	Cd	Pb
	[Hmim]Cl / [TEA][HSO_4]	0.074	0.376	0.093	0.013	0.007	0.538	0.004	0.005
	[Hmim]Cl / [DMBA][HSO_4]	0.018	0.054	0.082	0.012	0.007	0.256	0.003	0.012
	[TEA][HSO_4] / [DMBA][HSO_4]	0.241	0.357	0.765	0.692	0.709	0.199	0.600	0.362
(d)	Sludge	P	Cr	Ni	Cu	Zn	As	Cd	Pb
	STC / SWD	0.015	0.558	0.123	0.057	0.072	0.038	0.531	0.608
	STC / SWC	0.319	0.140	0.907	0.918	0.123	0.798	0.084	0.410
	SWD / SWC	0.068	0.366	0.178	0.010	0.001	0.034	0.950	0.334

Table S4: P-values from t-test performed on data from Figure 3 (main text). Metal extraction from (a) Severn Trent cake when treated with [Hmim]Cl for 45 min with S/L=0.1 at temperatures 25-120 °C; (b) Severn Trent cake when treated with [Hmim]Cl at 120 °C for 15-60 min with S/L=0.1; and (c) Severn Trent Cake when treated with [Hmim]Cl at 120 °C for 45 min for S/L loadings from 1/10 to 1/1 (w/w).

	**Variable**	**Metals**							
	Temperature	P	Cr	Ni	Cu	Zn	As	Cd	Pb
(a)	RT /30 ^{0}C	0.073	1.000	1.000	0.624	0.963	0.153	0.825	0.656
	RT /60 ^{0}C	0.203	1.000	1.000	0.182	0.209	0.774	0.187	0.174
	RT /90 ^{0}C	0.100	1.000	0.004	0.027	0.005	0.009	0.005	0.003
	RT /120 ^{0}C	0.027	0.075	0.002	0.016	0.002	0.004	0.002	0.001
	30 ^{0}C /60 ^{0}C	1.000	1.000	1.000	0.020	0.008	0.242	0.032	0.137
	30 ^{0}C /90 ^{0}C	0.756	1.000	0.001	0.006	0.000	0.002	0.000	0.001
	30 ^{0}C /120 ^{0}C	0.000	0.019	0.000	0.003	0.000	0.001	0.000	0.000
	60 ^{0}C /90 ^{0}C	0.896	1.000	0.002	0.031	0.002	0.041	0.001	0.005
	60 ^{0}C /120 ^{0}C	0.012	0.205	0.001	0.013	0.001	0.016	0.000	0.001
	90 ^{0}C /120 ^{0}C	0.001	0.047	0.066	0.489	0.119	0.252	0.006	0.037
(b)	Time (min)	P	Cr	Ni	Cu	Zn	As	Cd	Pb
	15/30	0.126	0.388	0.074	0.098	0.024	0.004	0.024	0.006
	15/45	0.523	0.280	0.343	0.380	0.029	0.011	0.023	0.019
	15/60	0.035	0.702	0.665	0.660	0.019	0.015	0.019	0.021
	30/45	0.056	0.200	0.163	0.524	0.925	0.635	0.926	0.784
	30/60	0.173	0.385	0.210	0.344	0.722	0.734	0.780	0.650
	45/60	0.018	0.882	0.829	0.728	0.815	0.940	0.855	0.882
(c)	S/L Loading	P	Cr	Ni	Cu	Zn	As	Cd	Pb
	0.1 / 0.2	0.150	0.262	0.243	0.112	0.313	0.212	0.174	0.216
	0.1 / 0.5	0.170	0.425	0.821	0.089	0.775	0.127	0.110	0.009
	0.1 / 1	0.170	0.177	0.011	0.003	0.064	0.004	0.003	0.005
	0.2 / 0.5	0.423	0.125	0.314	0.969	0.590	0.908	0.708	0.149
	0.2 / 1	0.695	0.317	0.482	0.009	0.649	0.104	0.069	0.018
	0.5 / 1	0.344	0.134	0.029	0.005	0.326	0.032	0.012	0.031

Table S5: P-values from t-test performed on data from Figure 4 (main text). (a) Extraction performance of recycled [Hmim]Cl on Severn Trent Cake over 6 cycles, (b) metal ion accumulation within the IL liquor over 6 cycles. Treatment was conducted at 120 °C for 45 min with S/L=0.1.

	**Variable**	**Metals**							
(a)	Cycle no.	P	Cr	Ni	Cu	Zn	As	Cd	Pb
	1 / 2	0.546	0.442	0.551	0.975	0.981	0.946	0.817	0.497
	1 / 3	0.102	0.146	0.157	0.078	0.092	0.042	0.030	0.201
	1 / 4	0.496	0.503	0.780	0.243	0.893	0.213	0.510	0.389
	1 / 5	0.132	0.295	0.124	0.171	0.132	0.122	0.011	0.123
	1 / 6	0.847	0.743	0.004	0.012	0.023	0.034	0.001	0.029
(b)	Cycle no.	P	Cr	Ni	Cu	Zn	As	Cd	Pb
	1 / 2	0.440	0.110	0.001	0.001	0.001	0.269	0.012	0.013
	2 / 3	0.001	0.001	0.001	0.001	0.001	0.062	0.000	0.000
	3 / 4	0.004	0.002	0.001	0.009	0.001	0.923	0.572	0.210
	4 / 5	0.024	0.231	0.035	0.027	0.048	0.615	0.096	0.040
	5 / 6	0.003	0.027	0.011	0.012	0.016	0.912	0.014	0.370
